# Idiopathic hypereosinophilia is clonal disorder? Clonality identified by targeted sequencing

**DOI:** 10.1371/journal.pone.0185602

**Published:** 2017-10-31

**Authors:** Jee-Soo Lee, Heewon Seo, Kyongok Im, Si Nae Park, Sung-Min Kim, Eun Kyoung Lee, Jung-Ah Kim, Joon-hee Lee, Sunghoon Kwon, Miyoung Kim, Insong Koh, Seungwoo Hwang, Heung-Woo Park, Hye-Ryun Kang, Kyoung Soo Park, Ju Han Kim, Dong Soon Lee

**Affiliations:** 1 Department of Laboratory Medicine, Seoul National University College of Medicine, Seoul, Republic of Korea; 2 Division of Biomedical Informatics, Seoul National University Biomedical Informatics (SNUBI), Seoul National University College of Medicine, Seoul, Republic of Korea; 3 Division of Biomedical Informatics, Systems Biomedical Informatics National Core Research Center, Seoul National University College of Medicine, Seoul, Republic of Korea; 4 Cancer Research Institute, Seoul National University College of Medicine, Seoul, Republic of Korea; 5 Department of Electrical Engineering, Seoul National University, Seoul, Republic of Korea; 6 Department of Laboratory Medicine, Hallym University Sacred Heart Hospital, Anyang, Republic of Korea; 7 Department of Physiology, College of Medicine, Hanyang University, Seoul, Republic of Korea; 8 Korean Bioinformation Center (KOBIC), Korea Research Institute of Bioscience and Biotechnology (KRIBB), Daejeon, Republic of Korea; 9 Department of Internal Medicine, Seoul National University College of Medicine, Seoul, Republic of Korea; Rutgers-Robert wood Johnson Medical School, UNITED STATES

## Abstract

Idiopathic hypereosinophilia (IHE)/idiopathic hypereosinophilic syndrome (IHES) has been defined by a persistent elevation of the blood eosinophil count exceeding 1.5×10^3^/μL, without evidence of reactive or clonal causes. While T-cell clonality assessment has been recommended for unexplained hypereosinophilia, this approach is not often applied to routine practice in the clinic. We hypothesized that the clonality would exist in a subset of IHE/IHES patients. We aimed to investigate the candidate mutations and T-cell clonality in IHE/IHES and to explore the role of mutations in eosinophil proliferation. We performed targeted capture sequencing for 88 genes using next-generation sequencing, T-cell receptor (TCR) gene rearrangement assays, and pathway network analysis in relation to eosinophil proliferation. By targeted sequencing, 140 variants in 59 genes were identified. Sixteen out of 30 patients (53.3%) harbored at least one candidate mutation. The most frequently affected genes were *NOTCH1* (26.7%), *SCRIB* and *STAG2* (16.7%), and *SH2B3* (13.3%). Network analysis revealed that our 21 candidate genes (*BIRC3*, *BRD4*, *CSF3R*, *DNMT3A*, *EGR2*, *EZH2*, *FAT4*, *FLT3*, *GATA2*, *IKZF*, *JAK2*, *MAPK1*, *MPL*, *NF1*, *NOTCH1*, *PTEN*, *RB1*, *RUNX1*, *TET2*, *TP53* and *WT1*) are functionally linked to the eosinophilopoietic pathway. Among the 21 candidate genes, five genes *(MAPK1*, *RUNX1*, *GATA2*, *NOTCH1* and *TP53*) with the highest number of linkages were considered major genes. A TCR assay revealed that four patients (13.3%) had a clonal TCR rearrangement. *NOTCH1* was the most frequently mutated gene and was shown to be a common node for eosinophilopoiesis in our network analysis, while the possibility of hidden T cell malignancy was indwelling in the presence of *NOTCH1* mutation, though not revealed by aberrant T cell study. Collectively, these results provide new evidence that mutations affecting eosinophilopoiesis underlie a subset of IHE/IHES, and the candidate genes are inferred to act their potential roles in the eosinophilopoietic pathway.

## Introduction

Idiopathic hypereosinophilia (IHE) is defined by persistent elevated eosinophils exceeding 1.5×10^3^/μL, satisfying an absence of causal underlying diseases. Diagnosis is based on the exclusion of reactive causes (i.e., parasitic infection; drug reaction; allergy and collagen vascular disorders) or clonal causes (i.e., chronic eosinophilic leukemia, not otherwise specified; myeloid-lymphoid neoplasms with eosinophilia associated with rearrangements of *PDGFRA*, *PDGFRB*, *FGFR1*, or *PCM1-JAK2* [1–4] and lymphocyte-variant hypereosinophilia) for eosinophilia. The patients presenting any organ damage due to infiltrated eosinophils are reclassified into idiopathic hypereosinophilic syndrome (IHES).

The current diagnostic criteria recommend the exclusion of lymphocyte-variant hypereosinophilia either by T-cell receptor (TCR) analysis or immunophenotyping [5], but consensus has not been formed [6]. Despite the criteria, approaches such as immunophenotyping or molecular TCR gene rearrangement studies are not often applied in routine practice, implying that a subset of IHE/IHES patients might have an underlying clonal state. Recent studies reported frequencies of somatic mutation ranging from 11% to 60% by application of next-generation sequencing (NGS) in IHE/IHES patients [7–9]. However, the biological role of the mutations in eosinophil proliferation remains to be determined.

**Fig 1:**
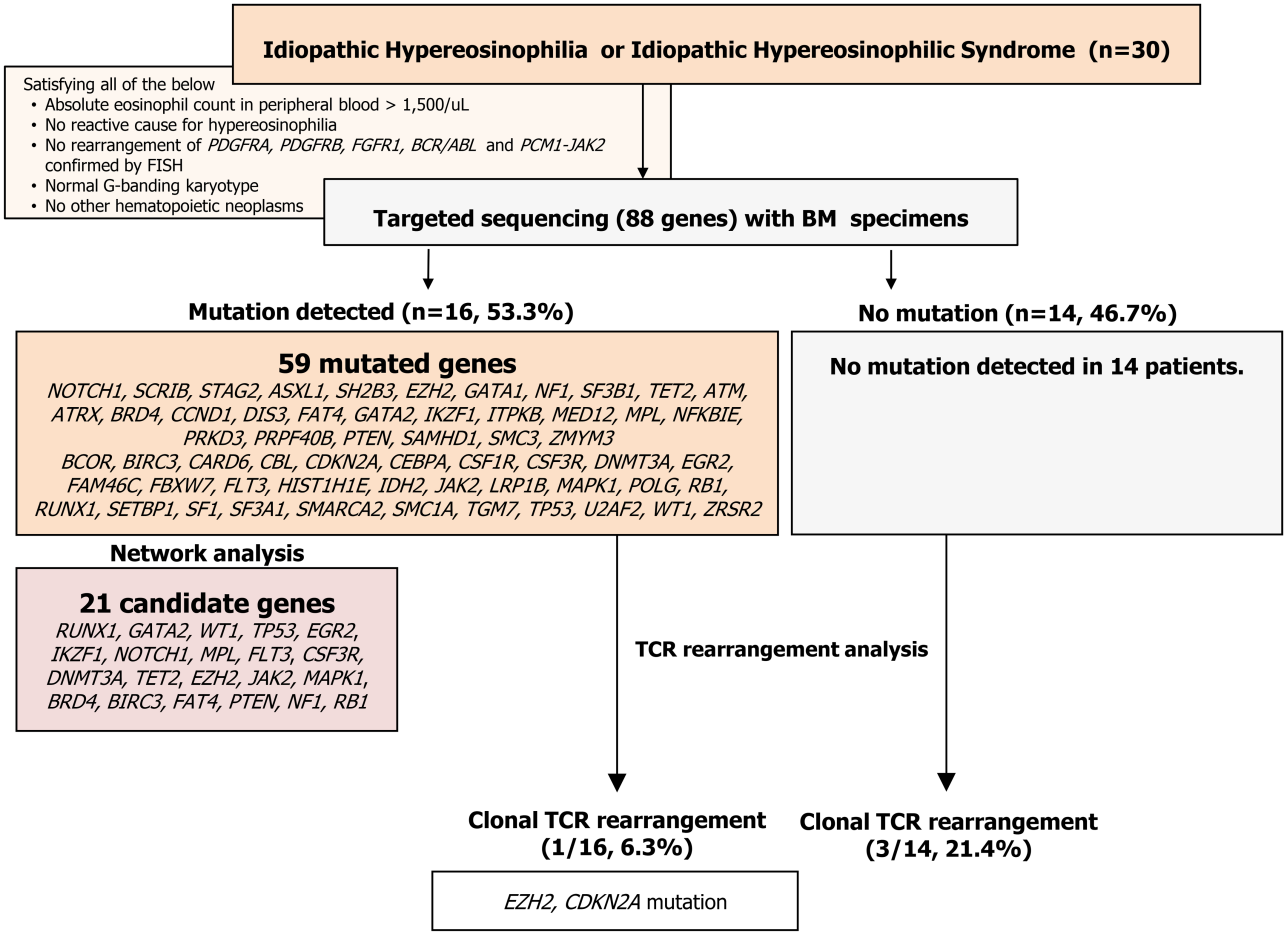
Flow of idiopathic hypereosinophilia study. Flow diagram showing how patients were included and evaluated in this study.

We hypothesized that a clonal nature might exist in patients classified as having IHE/IHES and that molecular markers might narrow the differential diagnosis of IHE/IHES. The aim of this study is to investigate the frequency of somatic mutations by NGS and hidden T-cell clonality by TCR gene rearrangement analysis in IHE/IHES and to explore the impact of the mutations on eosinophil proliferation. We performed targeted capture sequencing for 88 genes known to be involved in hematologic neoplasms along with a TCR gene rearrangement study. Furthermore, we tried to explore the impact of revealed mutations through pathway analysis in relevance to eosinophil proliferation. The flow chart of our study is presented in Fig 1.

## Materials and methods

### Patients

We evaluated 30 patients diagnosed with IHE or IHES between May 2004 and October 2014 at Seoul National University Hospital. The bone marrow (BM) samples were collected at the time of diagnosis or at a pre-treatment revisit. IHE/IHES was diagnosed strictly based on the diagnostic criteria of the World Health Organization [10]: 1) peripheral blood (PB) eosinophil count of >1.5×10^3^ /μL; 2) exclusion of reactive eosinophilia; and 3) exclusion of clonal eosinophilia and other hematopoietic neoplasms through the G-banding technique and fluorescent in situ hybridization (FISH) studies. The 30 IHE/IHES patients included 17 males and 13 females (median age, 47 years; range, 26–75). The following clinical information and laboratory results were obtained for each patient: sex; age of onset; the presence of hepatomegaly or splenomegaly; lymphadenopathy; constitutional symptoms; organ involvement, including cutaneous manifestations; complete blood cell counts; PB absolute eosinophil count; PB absolute lymphocyte count; serum IgE level; BM histological findings (eosinophil percentage, percentage of dysplastic eosinophils among 100 eosinophils counted, and cellularity); and clinical diagnosis (IHE or IHES). The clinical features and laboratory findings of the patients are summarized in S1 Table.

All BM samples were collected with informed consent, and the study was reviewed and approved by the Institutional Review Board of Seoul National University College of Medicine (IRB No. 1311-091-535).

### BM histological examination

Hematopathologists reviewed Wright-stained BM smears and hematoxylin-and-eosin-stained sections of BM trephine biopsies. The percentages of eosinophils, presence of blasts, and morphological dysplasia in each hematopoietic lineage were determined from the BM smears. The cellularity and infiltration of eosinophils in the BM sections were determined. For objective evaluation of eosinophil morphology, two hematopathologists conducted the morphology review, with the initial review conducted by one investigator and independently confirmed by the second investigator.

### Cytogenetic analysis and FISH

Cytogenetic studies using standard G-banding techniques were performed on heparinized BM samples as part of the diagnostic workup. Karyotypes were recorded according to the International System for Human Cytogenetic Nomenclature (ISCN) 2013 [11]. In all patients, interphase FISH analyses were performed on mononuclear cells of BM aspirates using the LSI *PDGFR*α, LSI *PDGFR*β, *FGFR1*, *BCR/ABL* (Vysis Inc., Downers Grove, IL, USA), *PCM1* (Empire Genomics, Buffalo, NY, USA) and *JAK2* (Cytocell, Cambridge, UK) probes. The FISH slides with BM cells were fixed with methanol:acetic acid (3:1), treated with 2× sodium saline citrate (SSC) for 30 minutes at 37°C, and dehydrated with 70%, 85% and 100% ethanol for 3 minutes each. A total of 10 μL of the probe mixture solution was placed onto the slides, and the slides were co-denatured at 75°C for 3 minutes. Then, the slides were hybridized overnight at 39°C in a humidified chamber. After hybridization, the slides were warmed in solution containing 0.4% SSC and 0.3% nonylphenol polyethylene glycol (NP-40) at 73°C for 2 minutes. Subsequently, the chromosomal DNA was counterstained with 6.6 μL of 4,6-diamidino-2-phenylindole dihydrochloride. The fluorescent signals were analyzed using a fluorescence microscope (Zeiss, Germany). A minimum of 200 cells in each specimen were assessed. The FISH results were recorded according to the ISCN 2013 guidelines [11]. The normal cut-off values for the deletion, amplification, or translocation of chromosomal regions were based on the means (± three standard deviations), and the binomial distribution function of 20 negative controls was analyzed.

### Targeted capture sequencing

To gain insight into the genetic lesions that leads to hypereosinophilia, we performed targeted capture sequencing of 88 hematologic neoplasm-related genes (S2 Table). Genomic DNA (gDNA) was extracted from the buffy coat of BM aspirates using the QIAamp DNA Blood Mini Kit (Qiagen, Valencia, CA, USA) according to the manufacturer’s instructions. The gDNA was sheared, the standard library was constructed and the hybridization step was performed at Celemics Inc. (Seoul, Korea). The final quality of the gDNA was assessed using an Agilent 2200 TapeStation System (Agilent, Santa Clara, CA, USA). We sequenced a total target length of 259 kb regions using the paired-end 150 bp rapid-run sequencing mode on an Illumina HiSeq 2500 platform (Illumina, San Diego, CA, USA) according to the manufacturer’s instructions. The sequencing data are uploaded to the Sequence Read Archive (SRA) (https://www.ncbi.nlm.nih.gov/sra) under accession number PRJNA398726.

### Variant filtering strategy

**Fig 2:**
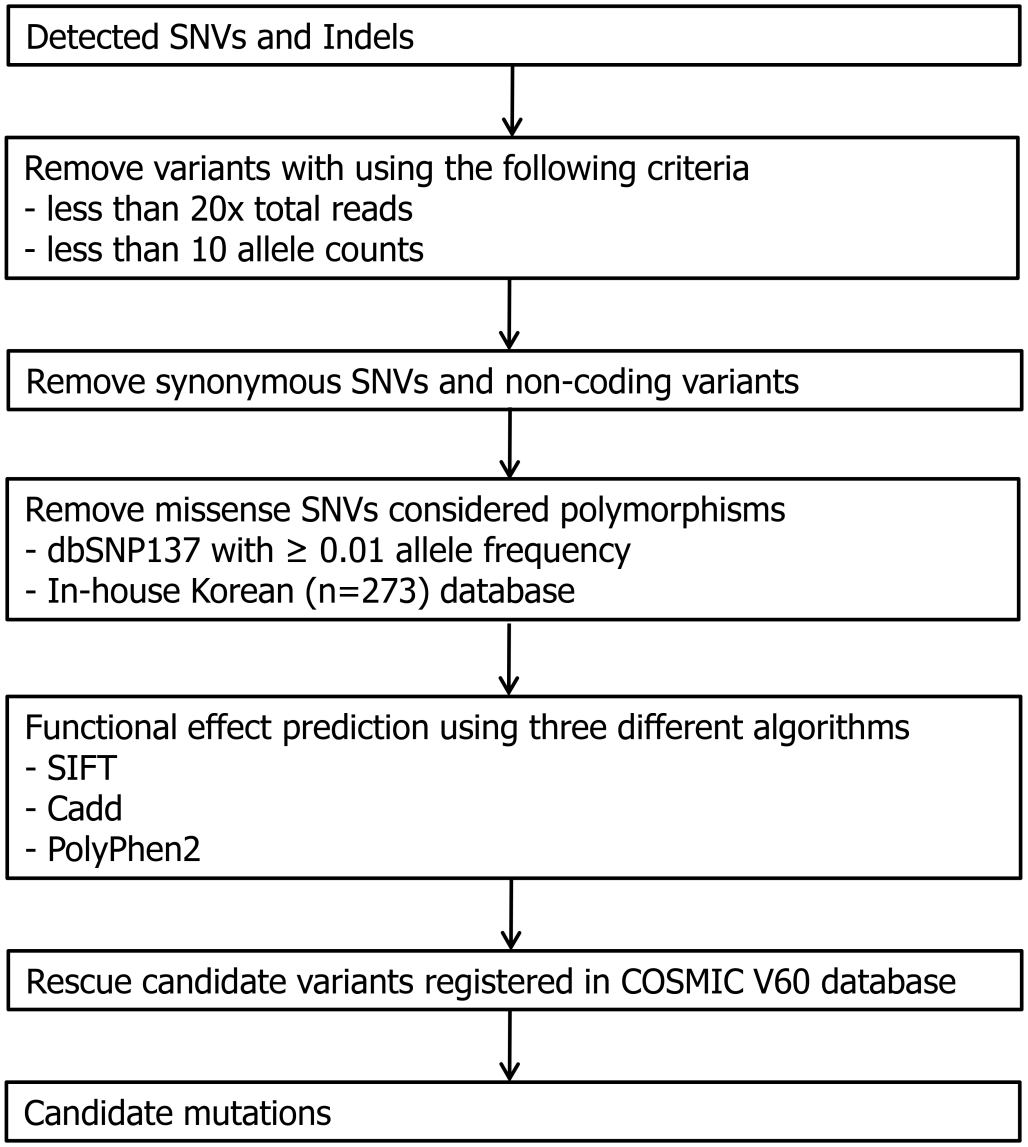
Workflow of filtering variants for the detection of candidate mutations. Flowchart shows the pipeline we used for filtering variants. Following exclusion of low quality variants (<20x total reads or <10 allele counts), synonymous, noncoding variants and polymorphisms were discarded. When recurrently reported in COSMIC V60 database, the variants were rescued.

The average coverage of the target regions was >800-fold. FASTQ files from the targeted capture sequencing results were aligned to the human reference genome (GRCh37) using the Burrows-Wheeler Aligner (BWA, v0.62) [12]. Duplicate PCR reads were removed using Picard 1.98, and variants were called using ‘Unified Genotyper’ in GATK 2.7–2 [13]. The stringent variant filtering strategy, applied for prioritizing candidate mutations, is presented in Fig 2. Briefly, variants with a low total depth (<20) and a low altered allele count (<10) were discarded to filter the low-quality variants. Synonymous and non-coding variants (i.e., intronic variants) were filtered out. Subsequently, the variants were excluded if they were within more than 0.01 allele frequencies, based on dbSNP137 [14]. Additionally, an in-house Korean single nucleotide polymorphism database was applied to filter out common variants in normal Korean controls (n = 273); single nucleotide variants identified in 273 Korean people were discarded. The functional effects of the missense variants were examined using in silico prediction algorithms: SIFT [15, 16], CADD [17] and PolyPhen2 [18]. Variants that were predicted to be deleterious using all three tools were included to minimize the false-positive rate. Variants recurrently presented in the COSMIC (v60) mutation database were rescued. All filtered variants were manually verified using Integrative Genomics Viewer [19, 20]. Detailed information describing our dataset is within the Supporting Information files.

### Network analysis

To assess the impact of the mutated genes on the mechanism of eosinophil proliferation, we performed a network analysis on the set of candidate genes and the 16 known eosinophilopoietic genes (*IL5*, *IL3*, *CSF2*, *ALOX5*, *C5*, *CCL5*, *CCL11*, *CCL13*, *CCL24*, *CCR3*, *LTB4R*, *PLA2G7*, *PTGDS*, *CEBPA*, *GATA1* and *SPI1*). The candidate genes and the known eosinophilopoietic genes were linked based on the published evidence provided by Pathway Studio (Elsevier, Atlanta, GA, USA). Each of the interactions was manually checked to ensure the biological interpretability of the network. We considered only the direct links from the candidate genes to the known genes and the undirected links between the two gene sets to focus on the regulatory effects of the candidate genes on the known genes. The network data were then exported to Cytoscape (http://www.cytoscape.org/) and visualized.

### TCR gene rearrangement assay

We performed the T-cell receptor beta (TCRB) gene rearrangement tests using IdentiClone, which is the EuroClonality/BIOMED-2 multiplex PCR assay (InVivoScribe Technologies, San Diego, CA, USA), according to the manufacturer’s recommendations. Because PCR assessment of clonal TCR is delicate and the interpretation is rather subjective, we used positive and negative controls along with known negative clinical specimens as controls in each run to minimize false-positive and false-negative interpretations. Unfortunately, we were unable to additionally analyze T-cell receptor gamma (TCRG) and T-cell receptor delta (TCRD) gene rearrangements due to a shortage of DNA samples. However, the majority of T-cell clones have a clonal TCRB rearrangement; a previous study reported that 91% (171/188) of T-cell malignancy cases showed clonal TCRB rearrangement [21, 22]. Clonality was analyzed using GeneMapper software version 3.0 (Life Technologies, Foster City, CA, USA) and interpreted according to the EuroClonality/BIOMED-2 guidelines [23, 24].

### Statistical analysis

Fisher’s exact test was used to compare categorical variables, and the Mann–Whitney U test was used for continuous variables. Pairwise correlations among gene mutations were calculated using Kendall's tau method. Statistical analyses were performed using SPSS version 19 (SPSS Inc., Chicago, IL, USA). *P*-values of <0.05 were considered statistically significant.

## Results

### Mutations characterized in hypereosinophilic patients

**Fig 3:**
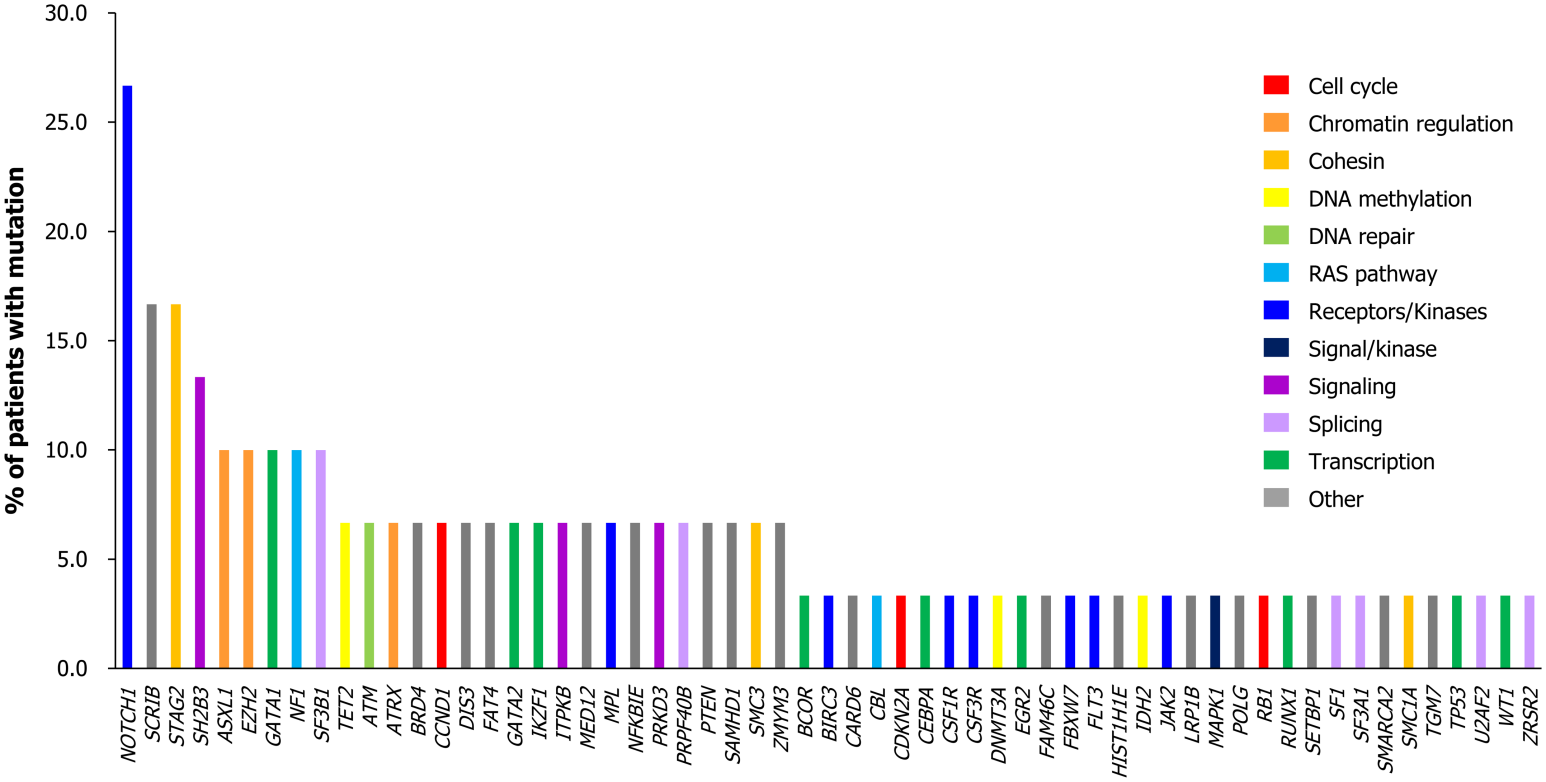
Distribution of mutated genes in idiopathic hypereosinophilia patients. The frequency of candidate mutations in each gene was listed for the all 16 patients with mutation positive.

**Fig 4:**
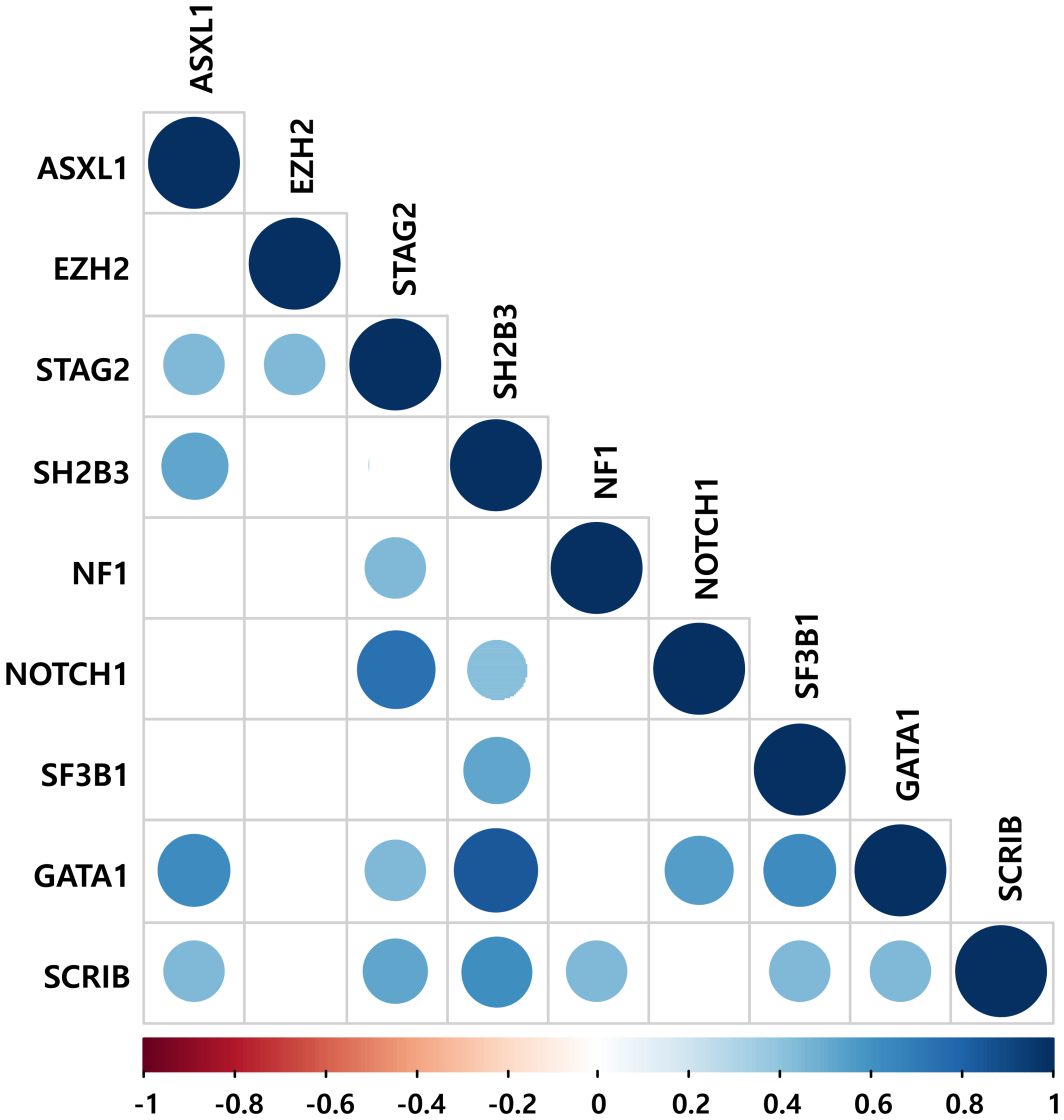
Correlations between frequently mutated genes (more than 2 patients). Statistically significant correlations (*P* < 0.05) were indicated. The correlation coefficients are shown by a color gradient and size difference.

A total of 140 candidate mutations in 59 genes were identified in 53.3% (n = 16) of the IHE/IHES patients; a median of one mutation [interquartile range (IQR), 0–3] per patient was confirmed (S3 Table). The most frequently affected genes were *NOTCH1*, *SCRIB*, *STAG2* and *SH2B3* (mutated in 26.7%, 16.7%, 16.7% and 13.3% of cases, respectively), followed by *ASXL1*, *EZH2*, *GATA1*, *NF1* and *SF3B1* (each gene was mutated in 10.0% of cases) (Fig 3). Among gene variants, positive correlations (*P* < 0.05) between *SH2B3*—*GATA1* (correlation coefficient, 0.850) and *NOTCH1*—*STAG2* (correlation coefficient, 0.742) mutations were observed (Fig 4). Additionally, *TP53*^V216M^, which was previously reported in a myeloproliferative neoplasm (MPN) case [25], was confirmed in a patient diagnosed with IHE (Case #21) (S3 Table).

### Network analysis

**Fig 5:**
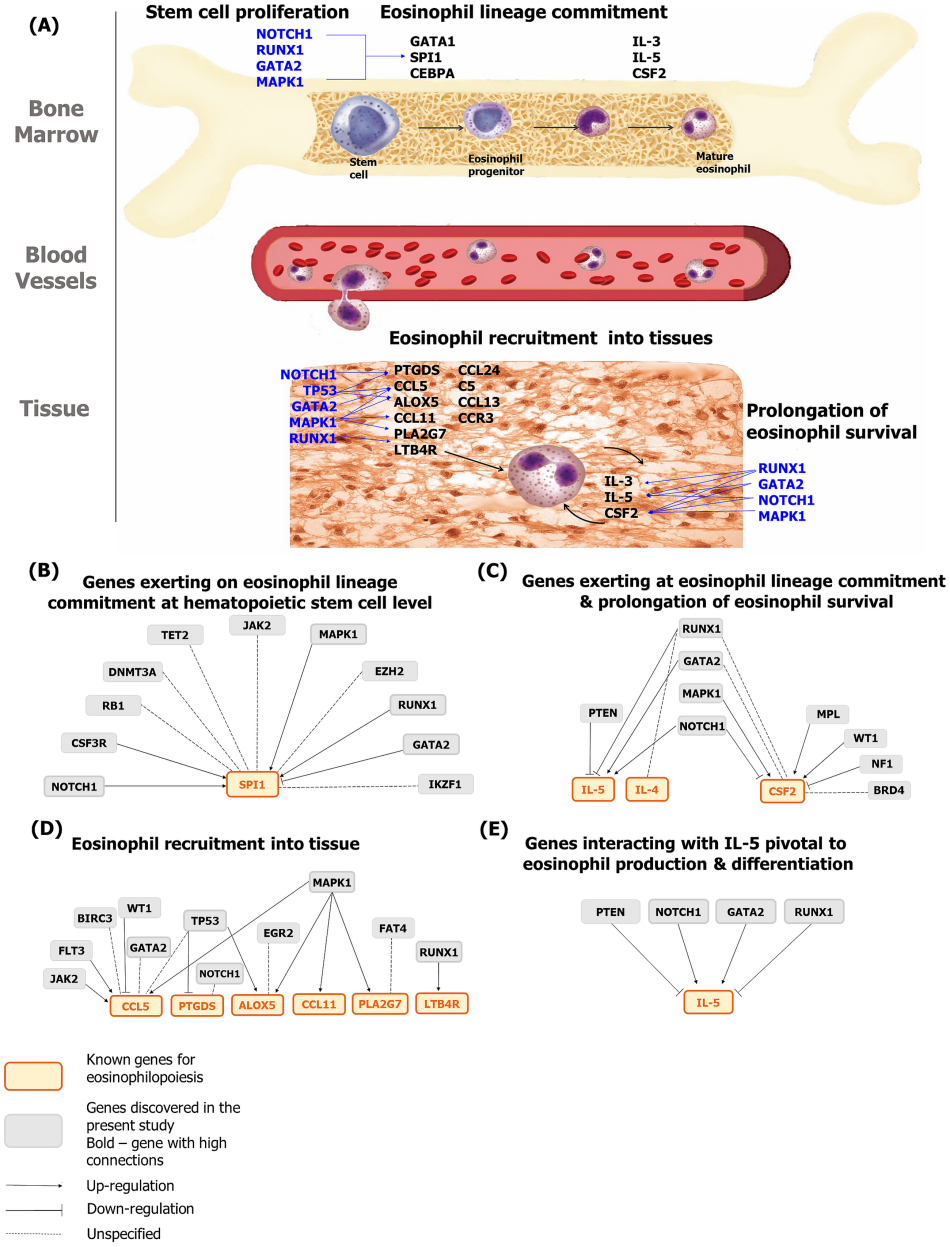
An illustration indicating action levels of discovered genes in the present study in relation to eosinophil production and Pathway Studio network analysis. Networks were created based on at least one published reference regarding candidate genes and the known 14 genes related to eosinophil production. (A) Genes marked with blue letter are well-known genes for which mechanisms are proven in eosinophil production. (B) Genes exerting on eosinophil lineage commitment at hematopoietic stem cell level. (C) Genes exerting at eosinophil lineage commitment and prolongation of eosinophil survival. (D) Eosinophil recruitment into tissue. (E) Genes interacting with IL-5, pivotal to eosinophil production and differentiation. *GATA1* and *CEBPA* were excluded from the network because they are involved in both eosinophil lineage commitment and the candidate gene set.

On the network analysis, 21 candidate genes were functionally linked to 10 known eosinophilopoietic genes, either at the gene or protein level (Fig 5 and S1 Fig). In Fig 5B–5E, five candidate genes (*MAPK1*, *RUNX1*, *GATA2*, *NOTCH1* and *TP53*) with the largest number of connections were shown with a thick gray border. Their high connectivity suggests their key regulatory role in eosinophil proliferation, making them the candidate genes of primary interest. The first four genes (*MAPK1*, *RUNX1*, *GATA2* and *NOTCH1)* are also linked to all three eosinophilic mechanisms (regulating eosinophil lineage specification, prolongation of eosinophil survival, and recruiting eosinophils into tissue), implying a multifaceted influence.

### Clonal T-cell population in hypereosinophilic patients

**Fig 6:**
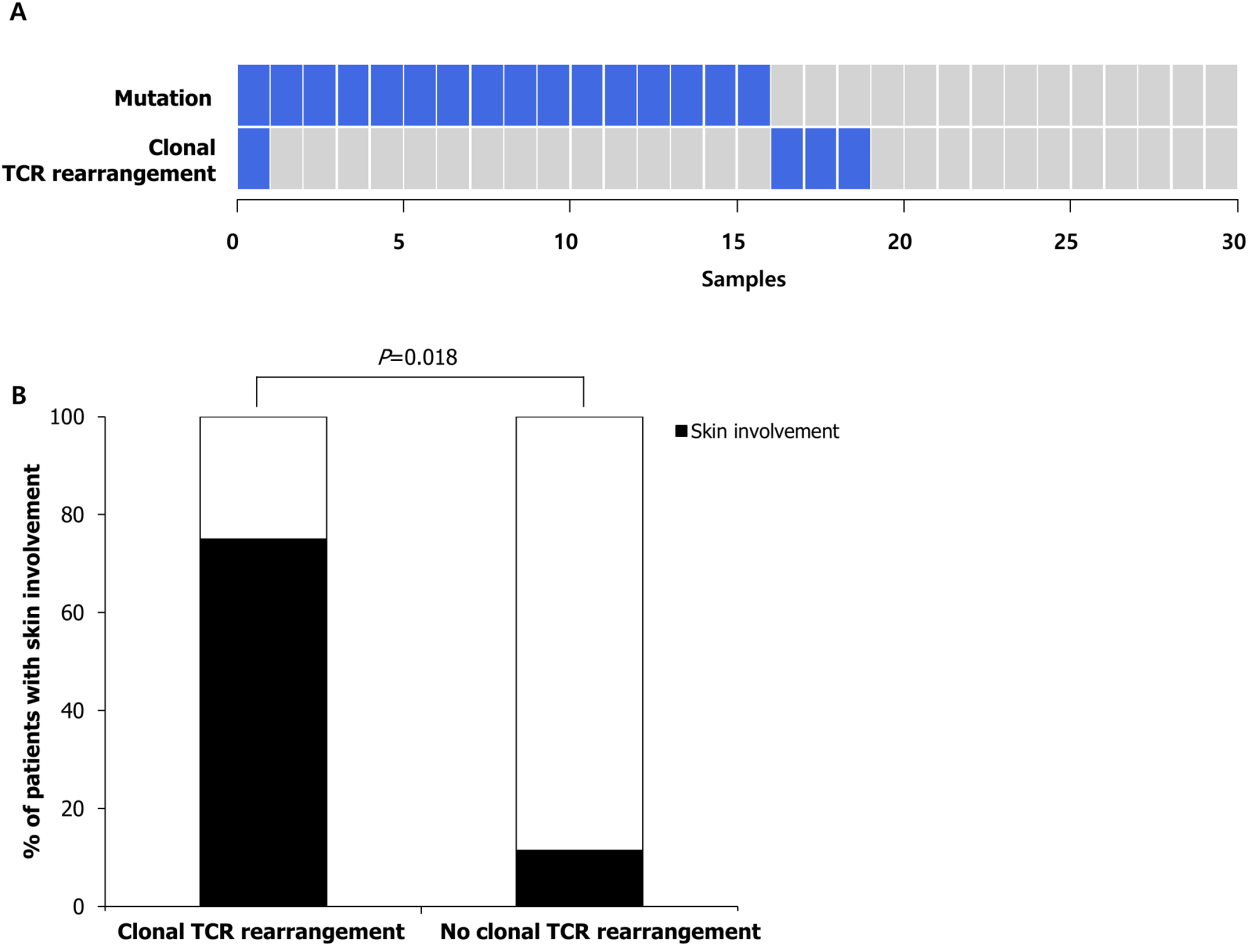
Mutation profiles and clinical features of patients with TCR rearrangement. (A) The incidence of mutations (n = 16) and clonal TCR rearrangements (n = 4) in IHE or IHES samples. Only one sample out of four clonal TCR rearrangement samples concurrently harbored somatic mutation. (B) Rate of skin involvement in IHE or IHES patients with or without clonal TCR rearrangement.

Four of 30 IHE/IHES cases showed clonal *TCRB* gene rearrangements (Fig 6A and S2 Fig). False-positive and false-negative findings were not detected in the controls. Clonal TCR rearrangements were mutually exclusive for the somatic mutations in all but one case. One patient (Case #3) with an aberrant T-cell population concurrently harbored mutations in *CDKN2A* and *EZH2* (Table 1). In patients with clonal TCR rearrangements, skin manifestation was more frequently observed than in patients without clonal TCR rearrangement (75% vs. 11.5%, *P* = 0.018) (Fig 6B).

**Table 1:** Mutated genes in patients with eosinophilia (n = 16).

Case ID	Gene
#2	*EZH2*, *FLT3*, *IKZF1*, *ITPKB*, *NOTCH1*, *SAMHD1*, *SF3A1*, *STAG2*, *ZMYM3*
#3*	*CDKN2A*, *EZH2*
#6	*ATRX*, *DIS3*, *NOTCH1*
#8	*NOTCH1*, *STAG2*
#9	*ATRX*, *BRD4*, *CARD6*, *GATA2*, *NFKBIE*, *SMC1A*
#10	*ASXL1*, *ATM*, *BIRC3*, *CBL*, *CCND1*, *CEBPA*, *DIS3*, *FAM46C*, *FAT4*, *FBXW7*, *GATA1*, *MAPK1*, *MPL*, *NF1*, *NFKBIE*, *NOTCH1*, *PRKD3*, *PRPF40B*, *RUNX1*, *SCRIB*, *SF1*, *SF3B1*, *SH2B3*, *SMC3*, *STAG2*, *TET2*, *WT1*
#12	*GATA1*, *NOTCH1*, *SF3B1*, *SH2B3*
#13	*SCRIB*, *SF3B1*
#14	*NF1*, *NOTCH1*, *PTEN*, *SCRIB*, *STAG2*
#15	*SCRIB*, *SH2B3*
#16	*MED12*, *NF1*
#17	*ASXL1*
#18	*ASXL1*, *ATM*, *BCOR*, *BRD4*, *CCND1*, *CSF1R*, *CSF3R*, *DNMT3A*, *EGR2*, *EZH2*, *FAT4*, *GATA1*, *GATA2*, *HIST1H1E*, *IDH2*, *IKZF1*, *ITPKB*, *JAK2*, *LRP1B*, *MED12*, *MPL*, *NOTCH1*, *POLG*, *PRKD3*, *PRPF40B*, *PTEN*, *RB1*, *SAMHD1*, *SCRIB*, *SETBP1*, *SH2B3*, *SMARCA2*, *SMC3*, *STAG2*, *TGM7*, *U2AF2*, *ZMYM3*, *ZRSR2*
#20	*NOTCH1*
#21	*TP53*
#28	*TET2*

* Clonal TCR rearrangement was detected in patient #3.

### Clinical features of IHE/IHES patients according to their somatic mutation status

**Fig 7:**
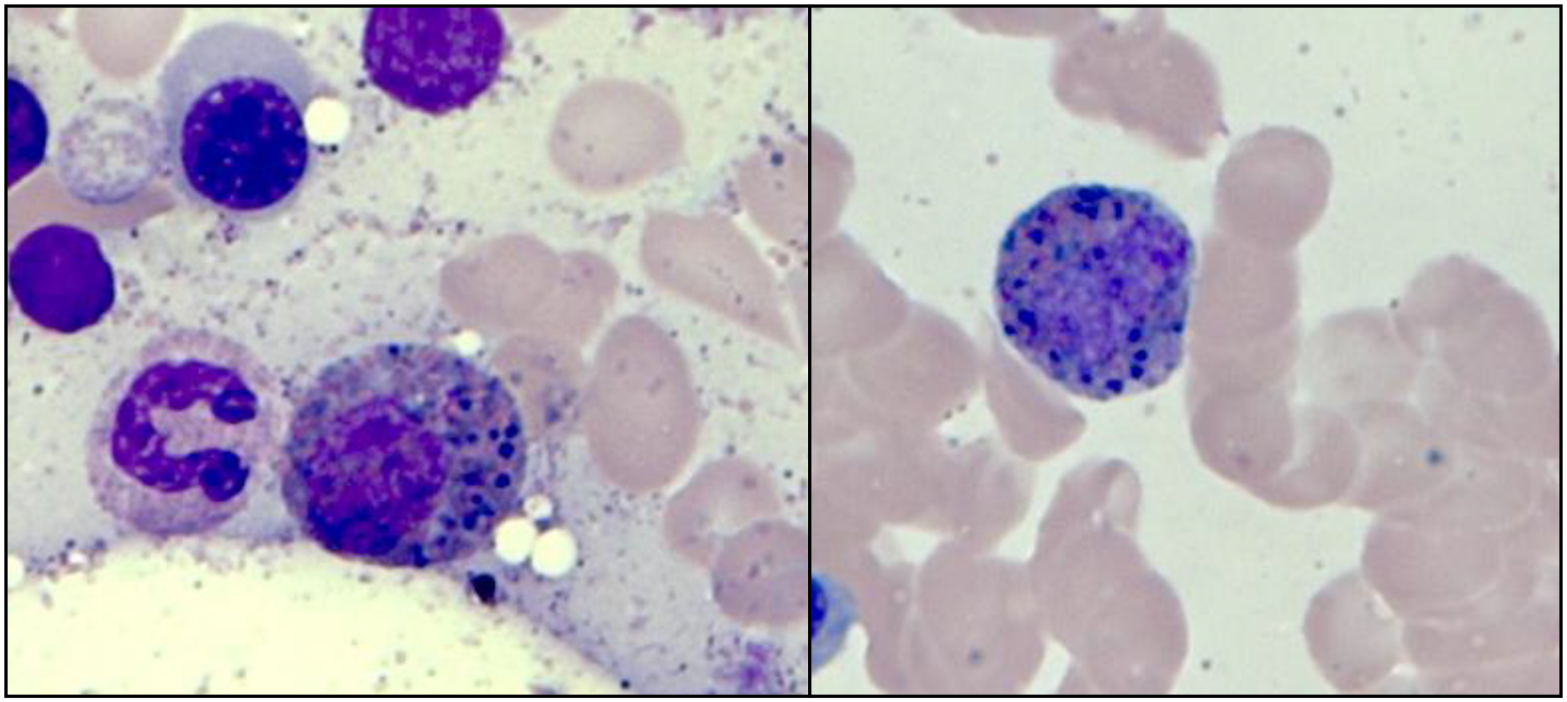
Dysplastic eosinophils frequently observed in IHE/IHES patients harboring mutations (n = 7). Cytoplasms are filled with abnormal secondary basophilic granules (BM, Wright-Giemsa, 1000×). Dysplastic eosinophils were more common in the mutation-positive group than in the mutation-negative group (*P* = 0.045).

We subdivided the 30 patients into two subgroups according to their results of targeted capture sequencing to examine the effect of the mutation status on the clinical manifestations: mutation positive (n = 16, carrying at least one candidate mutation) and mutation negative (n = 14, no evidence of harboring mutations). Dysplastic eosinophils, which were defined as eosinophils with abnormal secondary granules (basophilic color and larger-than-normal eosinophilic granules in the cytoplasm) were more frequently observed in the mutation-positive group (Fig 7). Half (53.3%, 7/16 cases) of the mutation-positive patients had dysplastic eosinophils in the BM (median of 2.0 per 100 total eosinophils, range, 0–23), while mutation-negative group had lower number of dysplastic eosinophils (median 0.3, range 0–5) (*P* = 0.045) (Table 2). Meanwhile, the other clinical characteristics (e.g., onset age, absolute eosinophil count, eosinophil percentages in the BM, the risk of end organ damage and constitutional symptoms) did not exhibit significant differences between the mutation-positive and mutation-negative subgroups.

**Table 2:** Patient clinical characteristics according to somatic mutation status.

	Patients with mutations (n = 16)	Patients without mutations (n = 14)	*P*-value
Onset age ^a^	44 (26–64)	51 (29–75)	ns
Male/Female (% male)	10/6 (62.5)	7/7 (50.0)	ns
CBC findings			
Hb (g/dL) ^a^	13.8 (8.2–15.4)	12.7 (8.6–15.1)	ns
WBC (×10^9^/L) ^a^	10.1 (4.12–50.4)	11.1 (6.0–38.1)	ns
Platelets (×10^9^/L) ^a^	159 (138–307)	252 (149–507)	ns
Peak AEC (×10^6^/L) ^a^	4,734 (851–44,463)	7,015 (2,580–24,365)	ns
Peak ALC (×10^6^/L) ^a^	2,071 (414–3,847)	2,122 (1,026–5,711)	ns
BM findings			
Eosinophils (%) ^a^	24.7 (2.0–82.8)	38.1 (9.0–66.0)	ns
Dysplastic eosinophils (n) ^a^^,^ ^b^	2 (0–23)	0.3 (0–5)	0.045
Erythroid dysplasia (%)	0 (0.0)	0 (0.0)	ns
Granuloid dysplasia (%)	0 (0.0)	0 (0.0)	ns
Megakaryocyte dysplasia (%)	0 (0.0)	0 (0.0)	ns
Granuloid hyperplasia (%)	2 (12.5)	3 (27.3)	
Hypercellular marrow (%)	0 (0.0)	1 (7.1)	ns
Splenomegaly (%)	1 (6.3)	0 (0.0)	ns
End organ damage (%)	10 (62.5)	12 (85.7)	ns
Constitutional symptom(s) (%)	7 (43.8)	9 (64.3)	ns
Treatment			
Corticosteroid (%)	10 (62.5)	13 (92.9)	ns
Hydroxyurea (%)	5 (31.3)	5 (35.7)	ns
Imatinib (%)	4 (25.0)	2 (14.3)	ns
IFN-alpha (%)	0 (0.0)	1 (7.1)	ns
Observation (%)	6 (37.5)	1 (7.1)	ns

Hb, hemoglobin; WBC, white blood cell; AEC, absolute eosinophil count; ALC, absolute lymphocyte count; BM, bone marrow; IFN, interferon; ns, not significant.

^a^. Age and laboratory values are presented as the medians (range).

^b^. The number of dysplastic eosinophils per 100 eosinophils was counted. The counts were estimated by two hematopathologists, and the average count was recorded.

## Discussion

The present study revealed that somatic mutations affecting hematopoietic cells are present in a subset of IHE/IHES patients and that these mutations are likely to be related to the clonal proliferation of eosinophils by pathway network analysis. Overall, 53.3% of IHE/IHES patients harbored somatic mutations; *NOTCH1*, *SCRIB*, *STAG2* and *SH2B3* mutations frequently occurred in 8 (26.7%), 5 (16.7%), 5 (16.7%) and 4 (13.3%) patients, respectively. Furthermore, *NOTCH1* and *SH2B3* mutations were more likely to coexist with *STAG2* and *GATA1* mutations, respectively. Currently it is unclear how the positive correlations between the genes noted above have synergistic effects on eosinophilia pathogenesis. Because the coexistence of a driver clone and the acquisition of additional mutations have been identified repeatedly in hematologic malignancies, further studies are required to elucidate how *SH2B3*—*GATA1* and *NOTCH1*—*STAG2* mutations contribute to the pathogenesis of hypereosinophilia.

*NOTCH1* was the most frequently mutated gene in this study, which implies a possible role for this gene in eosinophil differentiation. *NOTCH1* is also known as the notorious gene in T-cell malignancies. Activated forms of *NOTCH1* mutations, which are under the control of the *TCRB* locus, have been suggested to be the essential feature in T-cell acute lymphoblastic leukemia (T-ALL) pathogenesis [26]. In the present study, patients with *NOTCH1* mutations showed an absence of clonal TCR rearrangement, and there was no mutation overlap between previously reported T-ALL and our cases. However, considering that the TCR rearrangement study cannot catch all the cases of T-cell malignancies (sensitivity 91%, specificity 98%) [22], the possibility of hidden T-cell malignancies cannot be ruled out completely. Otherwise, as our network analysis revealed, *NOTCH1* mutations can explain an enhanced production of eosinophilia through a multifaceted role: regulating the cytokines that induce eosinophil lineage commitment and prolong eosinophil survival (IL-5 and CSF2), regulating the proteins that induce eosinophil tissue migration (PTGDS), and affecting hematopoietic stem cell level for eosinophil lineage commitment (SPI1).

*SCRIB* has been recently reported as a recurrently mutated gene in MPNs [27]. STAG2 forms a large ring-shaped cohesion complex together with SMC1A, SMC3 and RAD21 [28], and the integrity of this complex guarantees accurate homologous recombination in DNA repair [29]. *STAG2* mutations have been described in various tumor types: bladder cancer, glioblastoma, melanoma, Ewing’s sarcoma and myeloid malignancies [29]. *SH2B3* mutations have been identified in a wide range of myeloid diseases, including MPNs and myelodysplastic/myeloproliferative neoplasms [30]. In particular, MPN patients were found to carry *SH2B3* mutations at a frequency of 6.1–25.0% in previous studies [31–33]. The loss of *SH2B3* function in regulating the JAK2-STAT signaling pathway is believed to promote MPN development [30].

The network analysis identified that *TP53* up-regulates *ALOX5* expression, which affects eosinophil recruitment. Interestingly, *TP53*^V216M^ was confirmed in an IHE patient. One previous study reported *TP53*^V216M^ in a progenitor colony at acute myeloid leukemia (AML) diagnosis following *MPL*-mutant MPN, which was associated with leukemic progression [25]. Since previous studies have reported cases of IHE/IHES that ultimately evolved into acute leukemia or MPNs [34–36], we infer that close monitoring of these patients is required.

We attempted to determine whether clonal TCR gene rearrangements exist in IHE/IHES patients. Four (13.3%) patients had clonal T-cell populations. Previous studies revealed that 14%-42.8% of IHE/IHES patients had detectable T-cell clones, which were higher proportions than were observed in this study [37, 38]. In the present study, we focused only on the *TCRB* locus; thus, clonal *TCRG* and *TCRD* gene rearrangements could not be estimated. Helbig *et al*. reported that the majority of T-cell clones showed clonal *TCRB* rearrangements (18/42 patients, 42.8%) in HES patients, whereas clonal rearrangements in the *TCRG* locus (n = 1) and *TCRD* locus (n = 2) were rare [38]. In patients with clonal TCR rearrangements, the frequency of skin manifestation was significantly higher than in patients without clonal TCR rearrangement, which is comparable to previous reports [38, 39]. Although serum interleukin-5 (IL-5) level was not measured in this study, it has been reported that abnormal T cells overproduce IL-5, which promotes the differentiation of eosinophils [40]. IHES with abnormal T lymphocytes generally exhibit an indolent disease course, but the progression to overt T-cell lymphoma may be ultimately diagnosed in 5%-25% of the patients [5]. Thus, when a patient presents with distinct skin lesions accompanied by hypereosinophilia, a workup for T-cell clones should be emphasized.

One patient with clonal TCR rearrangement carried concurrent *CDKN2A* and *EZH2* mutations, suggesting two possibilities: 1) the presence of clonal *TCRB* gene rearrangements is not always equivalent to T-cell malignancy because non-neoplastic diseases (benign monoclonal γ-globulin disease, immunodeficient diseases associated with Epstein-Barr virus infections or autoimmune diseases) may also exhibit the clonal peak pattern [41, 42]; and 2) clonal TCR rearrangements with somatic mutations may suggest early stages of T-cell malignancy. Inactivation of the tumor suppressor gene *CDKN2A* has been shown to impair cell cycle arrest in multiple tumors, including T-ALL [43]. A loss of function *EZH2* mutation, which is well known to contribute to malignant hematopoiesis, was also reported in T-cell malignancy [43]. Because a previous study indicated that a few cases of TCR clonality with persistent hypereosinophilia have progressed to malignant T-cell diseases, the ‘high risk’ patients with clonal T-cells in our study should be observed closely [38].

We confirmed higher percentages of dysplastic eosinophils in the mutation-positive group. Cells with mixed eosinophil-basophil granules have been described in MPN and AML with inv(16)(p13q22) [44, 45]. These findings suggest that bi-granulated eosinophils may imply genetic instability, affecting granulocyte differentiation during hematopoiesis [45].

Several recent studies on IHE/IHES reported a wide range of mutation frequencies (11%-60%). The mutation frequency (53.3%) identified in the current study is higher than the frequency described in the previous two reports (11% and 28%), which applied targeted NGS-based panels covering 23 and 45 genes, respectively [7, 9]. Interestingly, Andersen *et al*. performed whole-exome sequencing for detecting somatic mutations in IHES and reported that mutations were detected in 60% of IHES patients [8]. We infer that the frequency of mutations might depend on the number of genes that the target gene panel covers.

While our results showed that 53.3% of IHE/IHES patients harbor somatic mutations related to hematologic neoplasms, most of the mutation sites were not previously reported in hematologic malignancies. Newly identified mutations are not SNP sites, and the significance is predicted as deleterious. Ultimately, additional functional studies are required to further clarify the biological roles of the specific mutations.

Our study has some limitations due to the retrospective study approach. First, the germline DNA of the patients was not evaluated in this study. To overcome this limitation, we applied stringent criteria to discriminate SNPs. We performed targeted sequencing analysis on 273 normal Korean controls and filtered variants that were present in normal Korean controls (Korean Mutation Database), and common variants on 1000 genome projects with more than 5% of allele frequency were filtered out. Another limitation of this study is that targeted capture sequencing was not performed at the single-cell level. Single-cell sequencing on each cell lineage (e.g., eosinophil and myeloid lineage) could be further performed in the future, which would enable the access to mutation status of each cell lineage and provide a comprehensive genetic landscape of IHE/IHES.

Despite such limitations, the result of this study contributes to identify the putative candidate genes and clonal T-cell populations in IHE/IHES. In addition, a primary strength of this study is the network analysis combined with targeted capture sequencing, which highlighted the candidate gene mutations in terms of their potential biological roles in the eosinophilopoietic pathway. Further large prospective studies are anticipated to confirm these findings.

In conclusion, this study strongly suggest that somatic mutations affecting hematopoietic cells underlie in a subset of IHE/IHES, and that these mutations are more likely to be associated with the clonal proliferation of eosinophils, possibly including MPN features. The presence of clonal mutations affecting hematopoietic stem cells or eosinophil differentiation in IHE/IHES may modify the concept of ‘idiopathic hypereosinophilia’. Either, somatic mutations in IHE/IHES reflect merely clonal eosinophilia of indeterminate potential (CEIP), similar to clonal hematopoiesis of indeterminate potential (CHIP). IHE/IHES with CEIP might carry high risk of developing T cell malignancies or MPN, as if individual with CHIP carries high risk of developing hematologic malignancies. Thus, patients harboring the underlying mutations should be closely followed up and monitored for the development of hematological malignancies.

## Supporting information

S1 Fig Pathway Studio network analysis (original version). The candidate genes and the known eosinophilopoietic genes were linked based on the published evidence provided by Pathway Studio.
(TIF)

S2 Fig Clonal T-cell receptor beta (TCRB) gene rearrangements observed in four patients (A-D). The clonal peak in each of the four patients was observed at 189 bp (A), 187 bp (B), 191 bp (C), and 189 bp (D), respectively.
(TIF)

S1 Table Clinical features and laboratory results of 30 IHE/IHES patients. (DOCX)

S2 Table Gene panel for targeted capture sequencing. (DOCX)

S3 Table Candidate mutations in the idiopathic hypereosinophilic patients (n = 16). (DOCX)
