# Supplementary figures and images for: Idiopathic hypereosinophilia is clonal disorder? Clonality identified by targeted sequencing
Source: PLoS One. 2017 Oct 31;12(10):e0185602. doi: 10.1371/journal.pone.0185602 (PMC5663336; doi:10.1371/journal.pone.0185602)

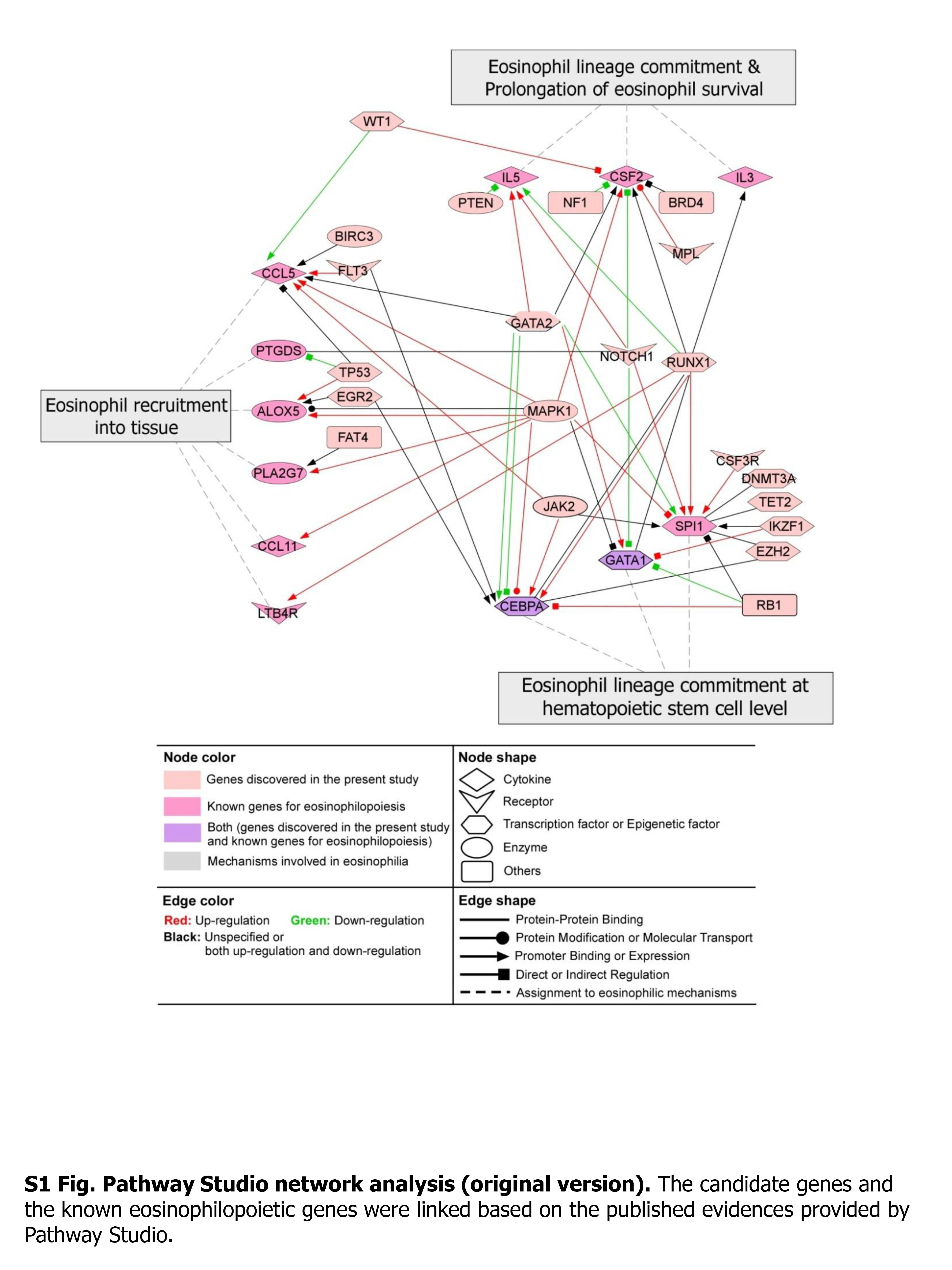

Supplement: S1 Fig — The candidate genes and the known eosinophilopoietic genes were linked based on the published evidence provided by Pathway Studio. (TIF) [file pone.0185602.s001.tif]

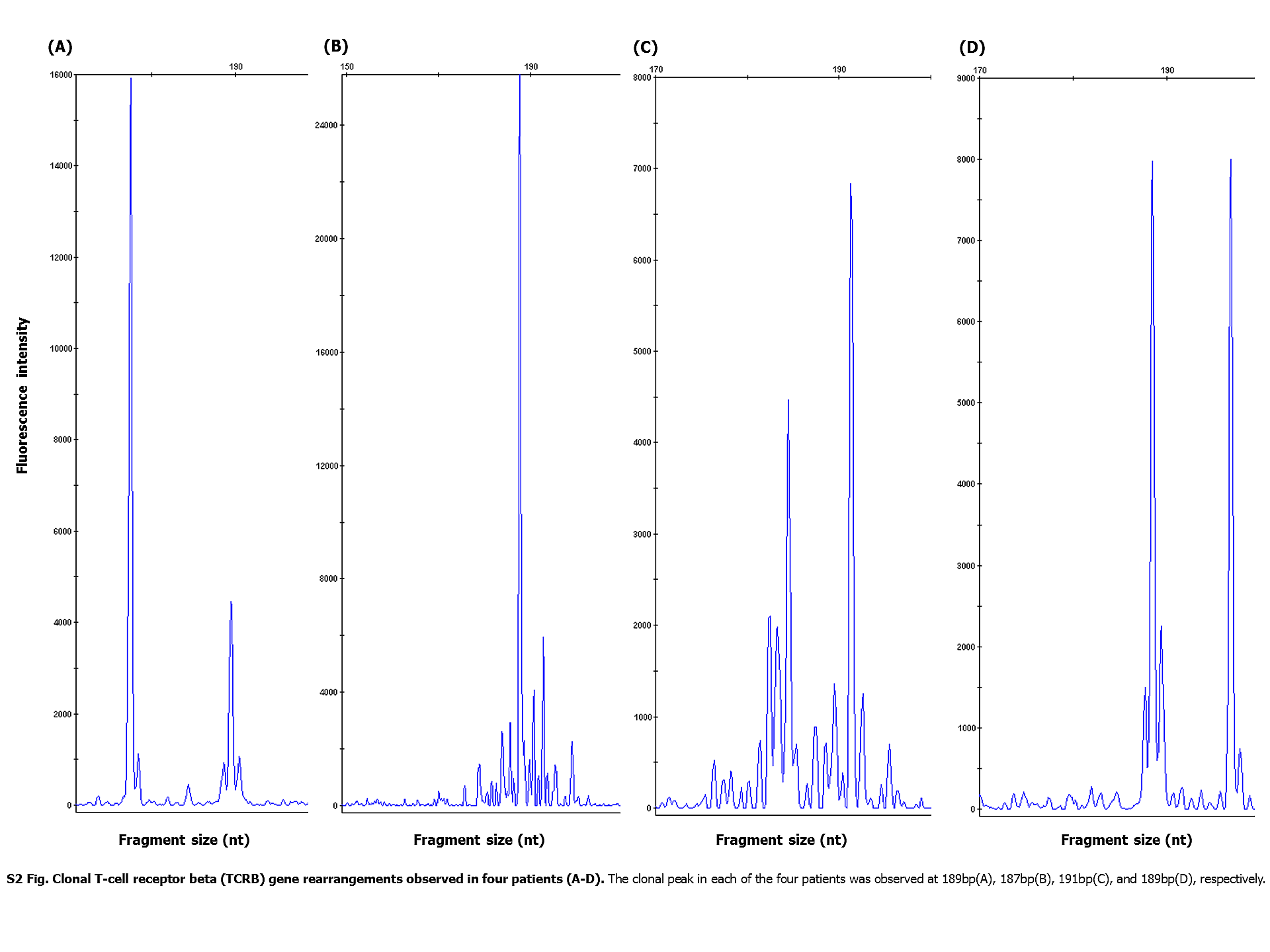

Supplement: S2 Fig — The clonal peak in each of the four patients was observed at 189 bp (A), 187 bp (B), 191 bp (C), and 189 bp (D), respectively. (TIF) [file pone.0185602.s002.tif]
