# Supplementary material for: Idiopathic hypereosinophilia is clonal disorder? Clonality identified by targeted sequencing
Source: PLoS One. 2017 Oct 31;12(10):e0185602. doi: 10.1371/journal.pone.0185602 (PMC5663336; doi:10.1371/journal.pone.0185602)
Supplement: S1 Table — (DOCX) [file pone.0185602.s003.docx]

**S1 Table**. Clinical features and laboratory results of 30 IHE/IHES patients.

| Case ID | Sex/  Age  of onset | HSM | LAP | Constitutional symptom(s) | Organ involvement | WBC (x10^9^/L) | Hb  (g/dL) | PLT (x10^9^/L) | AEC (x10^9^/L) | ALC (x10^9^/L) | Eo  (%) | Lymph (%) | IgE (IU/mL) | BM  Eo  (%) | BM  Dysplastic  Eo (%)^*^ | BM Cellularity (%) | Clinical diagnosis |
| --- | --- | --- | --- | --- | --- | --- | --- | --- | --- | --- | --- | --- | --- | --- | --- | --- | --- |
| #1 | M/47 | - | - | - | Liver | 17,500 | 15 | 295 | 19,179 | 3,938 | 58.4 | 22.5 | 22900 | 35.6 | 1.5 | 70 | IHES |
| #2 | M/31 | - | - | - | - | 9,250 | 14.1 | 220 | 4,440 | 1,600 | 44.6 | 17.3 | ND | 12.1 | 1.5 | 51-60 | IHE |
| #3 | M/59 | - | + | Dyspnea, Cough | Lung, skin | 10,080 | 13.7 | 277 | 11,350 | 1,391 | 50.8 | 13.8 | 249 | 19.0 | 0 | 51-60 | IHES |
| #4 | F/54 | - | - | - | - | 8,030 | 11.6 | 208 | 4,522 | 3,622 | 41.7 | 45.1 | ND | 15.0 | 0 | 31-40 | IHE |
| #5 | M/30 | - | - | Fever | Liver, lung | 15,930 | 14.4 | 175 | 7,992 | 2,182 | 27.1 | 13.7 | ND | 24.1 | 1.5 | 61-70 | IHES |
| #6 | F/27 | - | + | Pruritus,  Myalgia,  Joint pain | Skin | 12,960 | 11.3 | 287 | 10,458 | 1,153 | 69.6 | 8.9 | 175 | 31.7 | 0 | 41-50 | IHES |
| #7 | F/65 | - | - | Fatigue,  Polyneuropathy | Nervous system | 12,360 | 8.6 | 207 | 4,550 | 1,026 | 35.5 | 8.3 | 550 | 17.3 | 0 | 21-30 | IHES |
| #8 | F/64 | - | - | - | Liver | 14,150 | 13.6 | 260 | 5,027 | 3,679 | 19.6 | 26 | ND | 11.6 | 2.5 | 21-30 | IHES |
| #9 | M/61 | - | - | - | - | 8,330 | 14.5 | 293 | 1,599 | 2,624 | 19.2 | 31.5 | 213 | 9.8 | 4 | 31-40 | IHE |
| #10 | M/59 | - | - | - | Liver | 7,720 | 15.2 | 278 | 2,370 | 2,416 | 23.4 | 31.3 | 4850 | 16.9 | 3.5 | 21-30 | IHES |
| #11 | M/63 | - | - | Cough | Liver | 7,660 | 14.6 | 214 | 3,945 | 2,061 | 40.7 | 26.9 | 8500 | 21.6 | 7 | 41-50 | IHES |
| #12 | M/48 | - | - | - | - | 7,800 | 15.4 | 222 | 2,399 | 2,870 | 19.2 | 36.8 | ND | 12.4 | 1 | 41-60 | IHE |
| #13 | M/41 | - | - | Cough,  Abd pain,  Diarrhea | GI tract, liver, lung | 10,070 | 13.8 | 264 | 2,756 | 2,870 | 24 | 28.5 | 2490 | 19.9 | 23 | 0-10;  51-60 | IHES |
| #14 | M/54 | - | - | Cough | GI tract, liver, lung | 7,320 | 15.1 | 200 | 3,253 | 3,221 | 24 | 44 | 5000 | 10.3 | 3.5 | 41-50 | IHES |
| #15 | F/26 | - | - | Pruritus | Skin | 16,010 | 12.4 | 238 | 12,329 | 1,825 | 68.8 | 11.4 | 620 | 28.7 | 0 | 31-50 | IHES |
| #16 | F/55 | - | + | Fatigue,  Weight loss,  Cough,  Dyspnea | Nervoussystem, lung | 53,700 | 8.2 | 214 | 44,463 | 1,665 | 82.8 | 3.1 | ND | 54.9 | 0 | 51-60 | IHES |
| #17 | M/44 | Splenomegaly | - | - | - | 32,330 | 13.4 | 193 | 20,614 | 3,847 | 62.3 | 11.9 | ND | 38.2 | 8.5 | 41-60 | IHE |
| #18 | M/43 | - | - | Fatigue | Lung | 11,420 | 14.6 | 260 | 5,557 | 1,850 | 38.5 | 16.2 | 62 | 15.2 | 6.5 | 41-50 | IHES |
| #19 | M/36 | - | - | - | - | 9,750 | 14.3 | 308 | 6,037 | 1,784 | 43.1 | 18.3 | 3928 | 20.9 | 0 | 61-70 | IHE |
| #20 | F/40 | - | - | - | Liver | 4,120 | 11.7 | 140 | 851 | 2,225 | 10 | 54 | 113 | 4.6 | 8.5 | 41-50 | IHES |
| #21 | F/37 | - | - | - | - | 5,670 | 12.1 | 138 | 1,996 | 1,916 | 25.3 | 33.8 | 126 | 5.2 | 0 | 41-50 | IHE |
| #22 | M/55 | - | - | - | Skin | 7,540 | 13.8 | 264 | 8,457 | 2,051 | 30 | 27.2 | 24.3 | 18.8 | 0.5 | 41-50 | IHES |
| #23 | M/75 | - | + | Pruritus | Skin | 5,980 | 11.5 | 265 | 4,789 | 1,256 | 9 | 21 | 417 | 2.3 | 1 | 21-30 | IHES |
| #24 | M/63 | - | - | Fatigue | Skin | 6,810 | 15.1 | 149 | 2,994 | 2,520 | 24 | 37 | 434 | 20.3 | 1 | 41-50 | IHES |
| #25 | F/47 | - | - | Abd pain | Liver | 14,120 | 12.3 | 402 | 8,123 | 2,542 | 45 | 18 | 154 | 31.9 | 0.5 | 41-50 | IHES |
| #26 | F/46 | - | - | Dyspnea | Lung | 6,130 | 12.4 | 297 | 2,580 | 1,349 | 28.1 | 22 | ND | 5.7 | 0 | 41-50 | IHES |
| #27 | F/57 | - | - | - | Kidney | 12,930 | 9.7 | 507 | 11,279 | 2,974 | 28 | 23 | 8 | 29.0 | 0 | 31-40 | IHES |
| #28 | M/45 | - | - | - | - | 10,350 | 13.9 | 307 | 5,758 | 414 | 2 | 4 | 50 | 12.7 | 1 | 51-60 | IHE |
| #29 | F/37 | - | - | Fatigue,  Fever,  Myalgia | Lung | 38,070 | 9.8 | 171 | 24,365 | 5,711 | 64 | 15 | 133 | 66.2 | 0 | 71-80 | IHES |
| #30 | F/29 | - | - | Joint pain | Joint | 15,400 | 12.9 | 239 | 12,233 | 1,078 | 66 | 7 | 339 | 44.7 | 0 | 41-50 | IHES |

HSM, the presence of hepatomegaly or splenomegaly; LAP, lymphadenopathy; Abd, abdominal; GI tract, gastrointestinal tract; WBC, white blood cell; Hb, hemoglobin; PLT, platelet; Lymph, lymphocyte; AEC, absolute eosinophil count; ALC, absolute lymphocyte count; ND, not determined; BM, bone marrow; Eo, eosinophil; IHES, idiopathic hypereosinophilic syndrome; IHE, idiopathic hypereosinophilia.

* BM dysplastic eosinophils are expressed as percentages, i.e., the number of dysplastic eosinophils among 100 eosinophils counted.

Hypereosinophilia lasting for more than 6 months was not applied to our study criteria because the treatment was immediately started in most cases to minimize the eosinophil-induced tissue damage.
