# Supplementary material for: Idiopathic hypereosinophilia is clonal disorder? Clonality identified by targeted sequencing
Source: PLoS One. 2017 Oct 31;12(10):e0185602. doi: 10.1371/journal.pone.0185602 (PMC5663336; doi:10.1371/journal.pone.0185602)
Supplement: S2 Table — (DOCX) [file pone.0185602.s004.docx]

**S2 Table.** Gene panel for targeted capture sequencing

| Genes | NCBI Id. | Position | Exon count |
| --- | --- | --- | --- |
| *ASXL1* | 171023 | 20q11.1 | 18 |
| *ATM* | 472 | 11q22.3 | 69 |
| *ATRX* | 546 | Xq21.1 | 37 |
| *BARD1* | 580 | 2q35 | 13 |
| *BCOR* | 54880 | Xp11.14 | 18 |
| *BIRC3* | 330 | 11q22.2 | 11 |
| *BRAF* | 673 | 7q34 | 21 |
| *BRCC3* | 79184 | Xq28 | 12 |
| *BRD2* | 6046 | 6p21.3 | 17 |
| *BRD4* | 23476 | 19p13.1 | 23 |
| *CALR* | 811 | 9p13.3 | 9 |
| *CARD6* | 84674 | 5p13.1 | 4 |
| *CBL* | 867 | 11q23.3 | 18 |
| *CCND1* | 595 | 11q13.3 | 5 |
| *CDKN2A* | 1029 | 9p21 | 8 |
| *CEBPA* | 1050 | 19q13.1 | 1 |
| *CHD2* | 1106 | 15q26.1 | 39 |
| *CSF1R* | 1436 | 5q32 | 22 |
| *CSF3R* | 1441 | 1p34.3 | 19 |
| *DAP3* | 7818 | 1q22 | 15 |
| *DDX3X* | 1654 | Xp11.4 | 20 |
| *DIS3* | 22894 | 13q22.1 | 22 |
| *DNMT3A* | 1788 | 2p23 | 34 |
| *EEF1E1* | 9521 | 6p24.3 | 5 |
| *EGR2* | 1959 | 10q21.3 | 5 |
| *ETV6* | 2120 | 12p13.2 | 14 |
| *EZH2* | 2146 | 7q35-36 | 25 |
| *FAM46C* | 54855 | 1p12 | 2 |
| *FAT4* | 79633 | 4q28.1 | 18 |
| *FBXW7* | 55294 | 4q31.3 | 17 |
| *FLT3* | 2322 | 13q12 | 27 |
| *GATA1* | 2623 | Xp11.23 | 6 |
| *GATA2* | 2624 | 3q21.3 | 8 |
| *HIST1H1E* | 3008 | 6p22.2 | 1 |
| *IDH1* | 3417 | 2q33.3 | 12 |
| *IDH2* | 3418 | 15q26.1 | 12 |
| *IKZF1* | 10320 | 7p13 | 15 |
| *ITPKB* | 3707 | 1q42.12 | 10 |
| *JAK2* | 3717 | 9p24 | 26 |
| *KIAA0355* | 9710 | 19q13.11 | 16 |
| *KIT* | 3815 | 4q12 | 21 |
| *KLHL6* | 89857 | 3q27.1 | 7 |
| *KRAS* | 3845 | 12p12.1 | 8 |
| *LAMB4* | 22798 | 7q31.1 | 41 |
| *LRP1B* | 53353 | 2q21.2 | 92 |
| *MAPK1* | 5594 | 22q11.22 | 9 |
| *MED12* | 9968 | Xq13.1 | 45 |
| *MPL* | 4352 | 1p34.2 | 11 |
| *MYD88* | 4615 | 3p22.2 | 5 |
| *NF1* | 4763 | 17q11.2 | 62 |
| *NFKBIE* | 4794 | 6p21.1 | 6 |
| *NOTCH1* | 4851 | 9q34.3 | 34 |
| *NPM1* | 4869 | 5q35 | 11 |
| *NRAS* | 4893 | 1p13.2 | 7 |
| *PHF6* | 84295 | Xq26.2 | 10 |
| *PLEKHG5* | 57449 | 1p36.31 | 28 |
| *POLG* | 5428 | 15q25 | 23 |
| *POT1* | 25913 | 7q31.33 | 22 |
| *PRKD3* | 23683 | 2p22.2 | 21 |
| *PRPF40B* | 25766 | 12q13.12 | 28 |
| *PTEN* | 5728 | 10q23.3 | 10 |
| *PTPN11* | 5781 | 12q24.1 | 16 |
| *RAD21* | 5885 | 8q24.11 | 14 |
| *RB1* | 5925 | 13q14 | 28 |
| *RIPK1* | 8737 | 6p25.2 | 16 |
| *RUNX1* | 861 | 21q22.3 | 13 |
| *SAMHD1* | 25939 | 20q11.23 | 16 |
| *SCRIB* | 23513 | 8q24.3 | 37 |
| *SETBP1* | 26040 | 18q12.3 | 8 |
| *SF1* | 7536 | 11q13.1 | 16 |
| *SF3A1* | 10291 | 22q12.2 | 16 |
| *SF3B1* | 23451 | 2q33.1 | 27 |
| *SH2B3* | 10019 | 12q24.12 | 12 |
| *SMARCA2* | 6595 | 9p24.3 | 38 |
| *SMC1A* | 8243 | Xp11.22 | 26 |
| *SMC3* | 9126 | 10q25.2 | 29 |
| *SRSF2* | 6427 | 17q25.1 | 5 |
| *STAG2* | 10735 | Xq25 | 39 |
| *TCF12* | 6938 | 15q21.3 | 27 |
| *TET2* | 54790 | 4q24 | 11 |
| *TGM7* | 116179 | 15q15.2 | 14 |
| *TP53* | 7157 | 17p13.1 | 12 |
| *U2AF1* | 7307 | 21q22.3 | 9 |
| *U2AF2* | 11338 | 19q13.42 | 14 |
| *WT1* | 7490 | 11p13 | 11 |
| *XPO1* | 7514 | 2p15 | 28 |
| *ZMYM3* | 9203 | Xq13.1 | 26 |
| *ZRSR2* | 8233 | Xp22.1 | 14 |
