# Supplementary material for: Idiopathic hypereosinophilia is clonal disorder? Clonality identified by targeted sequencing
Source: PLoS One. 2017 Oct 31;12(10):e0185602. doi: 10.1371/journal.pone.0185602 (PMC5663336; doi:10.1371/journal.pone.0185602)
Supplement: S3 Table — (DOCX) [file pone.0185602.s005.docx]

**S3 Table.** Candidate mutations in the idiopathic hypereosinophilic patients (n=16)

| Gene | Chromosome | Position | Reference | Variant | Amino acid change | Variant classification | Insilico prediction  (SIFT\|Cadd\|PP2) | Read depth | Alternate allele fraction (%) | Case |
| --- | --- | --- | --- | --- | --- | --- | --- | --- | --- | --- |
| *ASXL1* | chr20 | 31022260 | C | A | Pro582His | missense | D\|D\|D | 379 | 12.66 | #18 |
| *ASXL1* | chr20 | 31022441 | A | AG | Gly646fs | frameshift | -\|-\|- | 777 | 12.61 | #10 |
| *ASXL1* | chr20 | 31022441 | A | AG | Gly646fs | frameshift | -\|-\|- | 1127 | 1.60 | #17 |
| *ASXL1* | chr20 | 31022441 | A | AG | Gly646fs | frameshift | -\|-\|- | 203 | 9.85 | #18 |
| *ASXL1* | chr20 | 31022824 | C | A | Ser770* | nonsense | -\|-\|- | 990 | 3.94 | #18 |
| *ASXL1* | chr20 | 31023440 | TC | T | Gln976fs | frameshift | -\|-\|- | 271 | 10.33 | #18 |
| *ATM* | chr11 | 108098506 | G | T | Glu26* | nonsense | -\|-\|- | 900 | 2.89 | #10 |
| *ATM* | chr11 | 108117703 | C | A | Ser305* | nonsense | -\|-\|- | 704 | 5.40 | #18 |
| *ATM* | chr11 | 108117790 | C | A | Ser334* | nonsense | -\|-\|- | 828 | 3.99 | #18 |
| *ATRX* | chrX | 76938311 | C | A | Glu813* | nonsense | -\|-\|- | 318 | 10.69 | #6 |
| *ATRX* | chrX | 76939520 | C | A | Glu410* | nonsense | -\|-\|- | 462 | 9.52 | #9 |
| *BCOR* | chrX | 39923784 | C | A | Glu1103* | nonsense | -\|-\|- | 188 | 14.89 | #18 |
| *BIRC3* | chr11 | 102206786 | G | T | Gly472* | nonsense | -\|-\|- | 1084 | 4.43 | #10 |
| *BRD4* | chr19 | 15355261 | G | A | Gln788* | nonsense | -\|-\|- | 42 | 52.38 | #9 |
| *BRD4* | chr19 | 15366298 | G | T | Ser619Arg | missense | D\|D\|D | 592 | 5.41 | #18 |
| *CARD6* | chr5 | 40852401 | G | T | Gly323* | nonsense | -\|-\|- | 6267 | 1.07 | #9 |
| *CBL* | chr11 | 119077304 | C | A | Cys59* | nonsense | -\|-\|- | 136 | 26.47 | #10 |
| *CCND1* | chr11 | 69456106 | G | T | Glu9* | nonsense | -\|-\|- | 44 | 40.91 | #18 |
| *CCND1* | chr11 | 69456242 | C | A | Pro54Gln | missense | D\|D\|D | 234 | 9.83 | #18 |
| *CCND1* | chr11 | 69457964 | G | T | Glu122* | nonsense | -\|-\|- | 121 | 19.01 | #18 |
| *CCND1* | chr11 | 69465895 | C | G | Arg245Gly | missense | D\|D\|D | 76 | 26.32 | #18 |
| *CCND1* | chr11 | 69466036 | G | T | Asp292Tyr | missense | D\|D\|D | 71 | 39.44 | #10 |
| *CDKN2A* | chr9 | 21971102 | C | T | Ala86Thr | missense | D\|D\|D | 154 | 12.99 | #3 |
| *CEBPA* | chr19 | 33792357 | C | A | Asp357Tyr | missense | D\|D\|NA | 210 | 16.67 | #10 |
| *CSF1R* | chr5 | 149441061 | C | T | Met617Ile | missense | D\|D\|D | 524 | 6.68 | #18 |
| *CSF3R* | chr1 | 36934796 | C | A | Gly513* | nonsense | -\|-\|- | 312 | 10.26 | #18 |
| *DIS3* | chr13 | 73337696 | C | A | Val674Phe | missense | D\|D\|D | 851 | 5.88 | #10 |
| *DIS3* | chr13 | 73346034 | C | A | Val502Phe | missense | D\|D\|D | 315 | 11.43 | #6 |
| *DNMT3A* | chr2 | 25458603 | T | C | Asp857Gly | missense | D\|D\|D | 1558 | 2.37 | #18 |
| *DNMT3A* | chr2 | 25462035 | G | A | Ala791Val | missense | D\|D\|D | 124 | 24.19 | #18 |
| *EGR2* | chr10 | 64575624 | C | A | Gly56* | nonsense | -\|-\|- | 238 | 7.98 | #18 |
| *EZH2* | chr7 | 148504755 | C | T | Glu747Lys | missense | D\|D\|D | 735 | 4.76 | #2 |
| *EZH2* | chr7 | 148506461 | C | T | Arg684His | missense | D\|D\|D | 136 | 16.18 | #18 |
| *EZH2* | chr7 | 148507478 | C | A | Arg659Ile | missense | D\|D\|D | 47 | 25.53 | #3 |
| *FAM46C* | chr1 | 118165974 | G | T | Gly162Trp | missense | D\|D\|D | 7368 | 0.76 | #10 |
| *FAT4* | chr4 | 126239037 | G | T | Gly491* | nonsense | -\|-\|- | 4443 | 1.15 | #10 |
| *FAT4* | chr4 | 126329688 | G | T | Gly1887Cys | missense | D\|D\|D | 1058 | 4.16 | #18 |
| *FAT4* | chr4 | 126370467 | G | T | Glu2768* | nonsense | -\|-\|- | 2316 | 2.59 | #10 |
| *FBXW7* | chr4 | 153271214 | G | T | Cys188* | nonsense | -\|-\|- | 648 | 5.86 | #10 |
| *FLT3* | chr13 | 28589333 | C | A | Gly905Val | missense | D\|D\|D | 703 | 5.12 | #2 |
| *GATA1* | chrX | 48649723 | C | G | Tyr69* | nonsense | -\|-\|- | 252 | 11.51 | #12 |
| *GATA1* | chrX | 48650801 | C | A | Leu224Ile | missense | D\|D\|D | 113 | 8.85 | #18 |
| *GATA1* | chrX | 48650816 | G | T | Gly229Cys | missense | D\|D\|D | 416 | 10.58 | #10 |
| *GATA2* | chr3 | 128202785 | C | A | Gly312Val | missense | D\|D\|D | 95 | 12.63 | #18 |
| *GATA2* | chr3 | 128202802 | C | A | Trp306Cys | missense | D\|D\|D | 101 | 15.84 | #18 |
| *GATA2* | chr3 | 128205856 | G | T | Gln7Lys | missense | D\|D\|D | 308 | 6.17 | #9 |
| *HIST1H1E* | chr6 | 26156853 | C | A | Arg79Ser | missense | D\|D\|D | 270 | 5.93 | #18 |
| *IDH2* | chr15 | 90633777 | C | A | Ala103Ser | missense | D\|D\|D | 127 | 10.24 | #18 |
| *IKZF1* | chr7 | 50467971 | C | A | Ser402Arg | missense | D\|D\|NA | 955 | 5.03 | #2 |
| *IKZF1* | chr7 | 50468300 | G | T | Gly512Val | missense | D\|D\|NA | 178 | 13.48 | #18 |
| *ITPKB* | chr1 | 226923854 | G | GC | Arg436fs | frameshift | -\|-\|- | 27 | 37.04 | #18 |
| *ITPKB* | chr1 | 226924961 | C | A | Glu67* | nonsense | -\|-\|- | 521 | 7.10 | #2 |
| *JAK2* | chr9 | 5090566 | G | A | Cys961Tyr | missense | D\|D\|D | 693 | 3.75 | #18 |
| *LRP1B* | chr2 | 141055448 | C | T | Cys4299Tyr | missense | D\|D\|D | 3023 | 11.38 | #18 |
| *LRP1B* | chr2 | 141641539 | C | A | Gly1339Val | missense | D\|D\|D | 139 | 25.90 | #18 |
| *MAPK1* | chr22 | 22127236 | G | T | Pro298Thr | missense | D\|D\|D | 851 | 4.94 | #10 |
| *MED12* | chrX | 70338631 | C | A | Tyr9* | nonsense | -\|-\|- | 331 | 9.97 | #16 |
| *MED12* | chrX | 70341430 | C | T | Gln289* | nonsense | -\|-\|- | 2054 | 13.39 | #18 |
| *MED12* | chrX | 70347196 | G | T | Val954Phe | missense | D\|D\|D | 1501 | 3.06 | #18 |
| *MED12* | chrX | 70349197 | C | A | Cys1203* | nonsense | -\|-\|- | 610 | 6.89 | #18 |
| *MED12* | chrX | 70354942 | A | T | Lys1622* | nonsense | -\|-\|- | 381 | 12.07 | #18 |
| *MPL* | chr1 | 43815028 | C | A | Tyr521* | nonsense | -\|-\|- | 274 | 5.11 | #18 |
| *MPL* | chr1 | 43815028 | C | G | Tyr521* | nonsense | -\|-\|- | 303 | 6.60 | #10 |
| *NF1* | chr17 | 29422329 | T | C | Met1? | start lost | -\|-\|- | 46 | 73.91 | #10 |
| *NF1* | chr17 | 29496972 | G | T | Gln181His | missense | D\|D\|D | 31 | 61.29 | #14 |
| *NF1* | chr17 | 29586074 | G | T | Val1453Phe | missense | D\|D\|D | 284 | 13.03 | #16 |
| *NFKBIE* | chr6 | 44227986 | C | T | Ala411Thr | missense | D\|D\|D | 190 | 8.42 | #10 |
| *NFKBIE* | chr6 | 44229482 | G | T | Ala330Asp | missense | D\|D\|D | 283 | 11.31 | #10 |
| *NFKBIE* | chr6 | 44233001 | G | T | Ser167Tyr | missense | D\|D\|D | 182 | 24.18 | #9 |
| *NOTCH1* | chr9 | 139393621 | C | A | Gly2009Cys | missense | D\|D\|D | 341 | 9.38 | #10 |
| *NOTCH1* | chr9 | 139396805 | C | A | Trp1768Leu | missense | D\|D\|D | 636 | 5.03 | #6 |
| *NOTCH1* | chr9 | 139401871 | C | A | Gly1177Trp | missense | D\|D\|D | 308 | 11.36 | #8 |
| *NOTCH1* | chr9 | 139402821 | C | A | Cys1063Phe | missense | D\|D\|D | 131 | 8.40 | #20 |
| *NOTCH1* | chr9 | 139403450 | C | A | Gly1015Cys | missense | D\|D\|D | 249 | 13.25 | #12 |
| *NOTCH1* | chr9 | 139404339 | C | T | Gly939Ser | missense | D\|D\|D | 134 | 14.93 | #18 |
| *NOTCH1* | chr9 | 139405620 | G | T | Cys857* | nonsense | -\|-\|- | 64 | 43.75 | #18 |
| *NOTCH1* | chr9 | 139417320 | C | A | Glu242* | nonsense | -\|-\|- | 29 | 68.97 | #10 |
| *NOTCH1* | chr9 | 139417467 | C | A | Gly193Cys | missense | D\|D\|D | 51 | 64.71 | #18 |
| *NOTCH1* | chr9 | 139417580 | C | A | Cys155Phe | missense | D\|D\|D | 277 | 15.16 | #14 |
| *NOTCH1* | chr9 | 139418192 | C | A | Cys127Phe | missense | D\|D\|D | 277 | 6.50 | #20 |
| *NOTCH1* | chr9 | 139418383 | G | T | Cys63* | nonsense | -\|-\|- | 67 | 19.40 | #10 |
| *NOTCH1* | chr9 | 139438506 | C | A | Cys37Phe | missense | D\|D\|D | 165 | 25.45 | #2 |
| *POLG* | chr15 | 89868894 | C | CG | Arg579fs | frameshift | -\|-\|- | 267 | 12.36 | #18 |
| *POLG* | chr15 | 89871965 | C | T | Arg374Gln | missense | D\|D\|D | 349 | 32.95 | #18 |
| *PRKD3* | chr2 | 37481387 | C | A | Arg820Leu | missense | D\|D\|D | 169 | 13.02 | #10 |
| *PRKD3* | chr2 | 37481427 | C | A | Asp807Tyr | missense | D\|D\|D | 126 | 12.70 | #10 |
| *PRKD3* | chr2 | 37494590 | C | A | Glu623* | nonsense | -\|-\|- | 526 | 6.46 | #10 |
| *PRKD3* | chr2 | 37496797 | C | A | Glu580* | nonsense | -\|-\|- | 668 | 5.54 | #10 |
| *PRKD3* | chr2 | 37516633 | G | A | Arg195* | nonsense | -\|-\|- | 5199 | 8.73 | #18 |
| *PRPF40B* | chr12 | 50028375 | C | A | Pro331Thr | missense | NA\|D\|D | 215 | 9.30 | #10 |
| *PRPF40B* | chr12 | 50031363 | G | T | Gln557His | missense | NA\|D\|D | 440 | 9.09 | #18 |
| *PRPF40B* | chr12 | 50037896 | C | A | Thr867Lys | missense | D\|D\|D | 3809 | 1.58 | #10 |
| *PTEN* | chr10 | 89653808 | G | T | Gly36* | nonsense | -\|-\|- | 479 | 9.19 | #18 |
| *PTEN* | chr10 | 89653856 | G | A | Asp52Asn | missense | NA\|D\|D | 503 | 8.75 | #18 |
| *PTEN* | chr10 | 89690811 | A | T | Glu73Val | missense | NA\|D\|D | 627 | 5.74 | #14 |
| *RB1* | chr13 | 49033835 | G | T | Ala658Ser | missense | D\|D\|D | 2496 | 2.36 | #18 |
| *RUNX1* | chr21 | 36164692 | G | T | Pro395Thr | missense | D\|D\|D | 90 | 25.56 | #10 |
| *SAMHD1* | chr20 | 35521341 | TG | T | Pro625fs | frameshift | -\|-\|- | 568 | 44.19 | #2 |
| *SAMHD1* | chr20 | 35579994 | C | A | Ser18Ile | missense | D\|D\|D | 482 | 6.64 | #18 |
| *SCRIB* | chr8 | 144874057 | G | T | Pro1562Thr | missense | D\|D\|D | 71 | 23.94 | #10 |
| *SCRIB* | chr8 | 144886964 | T | A | His928Leu | missense | D\|D\|D | 70 | 35.71 | #10 |
| *SCRIB* | chr8 | 144887569 | C | A | Glu795* | nonsense | -\|-\|- | 185 | 7.57 | #13 |
| *SCRIB* | chr8 | 144887569 | C | A | Glu795* | nonsense | -\|-\|- | 168 | 8.33 | #15 |
| *SCRIB* | chr8 | 144889096 | C | A | Glu756* | nonsense | -\|-\|- | 249 | 12.85 | #14 |
| *SCRIB* | chr8 | 144889134 | G | T | Ala743Glu | missense | D\|D\|D | 288 | 11.11 | #13 |
| *SCRIB* | chr8 | 144890842 | C | CAAGGT | Glu685fs | frameshift | -\|-\|- | 144 | 16.67 | #18 |
| *SETBP1* | chr18 | 42281603 | T | A | Phe98Ile | missense | D\|D\|D | 4683 | 1.15 | #18 |
| *SF1* | chr11 | 64537849 | C | A | Glu215* | nonsense | -\|-\|- | 536 | 6.72 | #10 |
| *SF3A1* | chr22 | 30736704 | C | T | Arg390His | missense | D\|D\|D | 900 | 4.78 | #2 |
| *SF3B1* | chr2 | 198263248 | A | C | Leu1024* | nonsense | -\|-\|- | 8103 | 0.67 | #10 |
| *SF3B1* | chr2 | 198266500 | G | T | Ser779Tyr | missense | D\|D\|D | 1342 | 3.43 | #12 |
| *SF3B1* | chr2 | 198267753 | C | A | Val576Leu | missense | D\|D\|D | 355 | 9.01 | #13 |
| *SH2B3* | chr12 | 111856529 | G | A\|T | Glu194* | nonsense | -\|-\|- | 139 | 14.39 | #15 |
| *SH2B3* | chr12 | 111884801 | C | A | Ser297* | nonsense | -\|-\|- | 296 | 10.14 | #10 |
| *SH2B3* | chr12 | 111884810 | C | A | Ala300Asp | missense | D\|D\|D | 215 | 7.44 | #12 |
| *SH2B3* | chr12 | 111885964 | TG | T | Val529fs | frameshift | -\|-\|- | 262 | 9.92 | #18 |
| *SMARCA2* | chr9 | 2056823 | G | T | Arg442Met | missense | D\|D\|D | 1735 | 2.65 | #18 |
| *SMC1A* | chrX | 53436051 | C | A | Arg496Leu | missense | D\|D\|D | 3008 | 1.60 | #9 |
| *SMC3* | chr10 | 112341722 | G | T | Glu197* | nonsense | -\|-\|- | 2608 | 1.69 | #10 |
| *SMC3* | chr10 | 112363993 | G | C | Ser1196Thr | missense | D\|D\|D | 399 | 9.27 | #18 |
| *STAG2* | chrX | 123181204 | C | A | Ala223Asp | missense | NA\|D\|D | 886 | 4.06 | #8 |
| *STAG2* | chrX | 123181281 | G | T | Glu249* | nonsense | -\|-\|- | 661 | 3.18 | #10 |
| *STAG2* | chrX | 123181281 | G | T | Glu249* | nonsense | -\|-\|- | 1527 | 4.06 | #18 |
| *STAG2* | chrX | 123182906 | G | T | Gly291* | nonsense | -\|-\|- | 493 | 6.49 | #10 |
| *STAG2* | chrX | 123190007 | A | G | Asp409Gly | missense | D\|D\|D | 847 | 4.72 | #18 |
| *STAG2* | chrX | 123195134 | A | T | Lys493* | nonsense | -\|-\|- | 1816 | 1.87 | #18 |
| *STAG2* | chrX | 123197020 | G | T | Asp596Tyr | missense | D\|D\|D | 139 | 8.63 | #2 |
| *STAG2* | chrX | 123227947 | G | T | Glu1220* | nonsense | -\|-\|- | 1353 | 3.40 | #14 |
| *TET2* | chr4 | 106156991 | C | A | Ser631* | nonsense | -\|-\|- | 11543 | 0.62 | #10 |
| *TET2* | chr4 | 106158550 | G | T | Glu1151* | nonsense | -\|-\|- | 1168 | 47.95 | #28 |
| *TET2* | chr4 | 106180811 | C | T | Thr1280Ile | missense | D\|D\|NA | 2025 | 2.42 | #10 |
| *TGM7* | chr15 | 43572000 | C | A | Glu501* | nonsense | -\|-\|- | 229 | 16.59 | #18 |
| *TGM7* | chr15 | 43574804 | C | A | Trp340Leu | missense | D\|D\|D | 179 | 8.38 | #18 |
| *TP53* | chr17 | 7578203 | C | T | Val216Met | missense | D\|D\|D | 212 | 46.70 | #21 |
| *U2AF2* | chr19 | 56175048 | G | A | Arg227His | missense | D\|D\|D | 637 | 21.98 | #18 |
| *U2AF2* | chr19 | 56185361 | G | T | Arg452Leu | missense | D\|D\|D | 401 | 17.71 | #18 |
| *WT1* | chr11 | 32456333 | G | T | Gln187Lys | missense | D\|D\|D | 317 | 6.62 | #10 |
| *ZMYM3* | chrX | 70462190 | G | T | Thr1211Asn | missense | D\|D\|D | 2163 | 1.02 | #2 |
| *ZMYM3* | chrX | 70472853 | G | GC | Leu85fs | frameshift | -\|-\|- | 154 | 17.53 | #18 |
| *ZRSR2* | chrX | 15840862 | G | T | Glu316* | nonsense | -\|-\|- | 132 | 33.33 | #18 |

D, deleterious; NA, not available

In silico tools were applied to predict the effect of the missense variants.
